# The effect of Total resistance exercise vs. aquatic training on self-reported knee instability, pain, and stiffness in women with knee osteoarthritis: a randomized controlled trial

**DOI:** 10.1186/s13102-020-00175-y

**Published:** 2020-04-29

**Authors:** Shirin Assar, Farzaneh Gandomi, Mahsa Mozafari, Freshteh Sohaili

**Affiliations:** 1 Clinical Research Development Center, Imam Reza Hospital, Kermanshah University of Medical Sciences, Kermananshah, Iran; 2 Department of Corrective Exercises and Sport Injuries, Faculty of Physical Education and Sport Sciences, Razi University, Kermanshah, Iran; 3 Department of Sport Biomechanics, Faculty of Physical Education and Sport Sciences, Bu-Ali-Sina University, Hamedan, Iran; 4 Department of Sport Physiology, Faculty of Physical Education and Sport Sciences, Razi University, Kermanshah, Iran

**Keywords:** Self-reported knee instability, Osteoarthritis, TRX, Aquatic exercises

## Abstract

**Background:**

Knee Instability (KI) is described as a sense of knee buckling, shifting, or giving way during the weight bearing activities. High prevalence (60–80%) has been reported for KI amongst the patients with knee osteoarthritis (KOA). In this line, the present study targeted the effect of two interventions on self-reported KI and affected factors.

**Methods:**

In this single blind, randomized, and controlled trial, 36 patients with radiographic grading (Kellgren–Lawrence ≥ II) of KOA were selected. Patients were divided into three groups namely, aquatic (*n* = 12), Total Resistance exercises (TRX) (n = 12) and control (n = 12) by random. Then both 8-week TRX and aquatic exercises were carried out by experimental groups. The following measure were taken before and after interventions: Pain by visual analog scale (VAS), balance by Berg Balance Scale (BBS), quadriceps strength by dynamometer, knee flexion range of motion (ROM) by inclinometer, knee stiffness with Western Ontario and McMaster Universities Osteoarthritis (WOMAC), and self-reported KI with Felson’s questionnaire.

**Results:**

The results demonstrated that KI, VAS, BBS improved over time both in TRX and aquatic groups significantly (*p* < 0.05), but WOMAC_(stiffness)_, knee flexion ROM, and quadriceps strength were significantly improved over time only for TRX (*p* < 0.05). Post hoc test, also, showed that there were significant differences between interventions and control groups (p < 0.05) for the VAS, KI, BBS, but for WOMAC_(stiffness),_ a significant difference was observed only between TRX and control groups (*p* = 0.05).

**Conclusions:**

Although TRX and aquatic interventions had a similar effect on the patients’ balance, pain and KI, TRX had more effect on WOMAC_(stiffness)_, quadriceps strength, and knee flexion ROM than aquatic exercises.

**Trial registration:**

This study was registered in the Iranian Clinical Trial Center with the number IRCT20181222042070N1, http://www.irct.ir/trial/36221, registered 02 February 2019.

## Background

Knee instability (KI) is the most common problem amongst patients with knee osteoarthritis (KOA) which can affect weight bearing or walking. Knee instability is described as a sense of the knee buckling, shifting, or giving way during the weight bearing activities. High prevalence rate (60–80%) has been reported for KI amongst the patients with KOA [1]. In prior studies, knee instability has been associated with pain increasing, activity daily living disrupting, gait pattern altering, and also fall number increasing [2, 3]. Additionally, investigations have reported that the factors involved in neuromuscular deficiency such as joint laxity, proprioception deficiency, and inappropriate muscle stiffness strategies can expose patients to this instability [3–6]. Accordingly, Schmitt and Rudolph (2007) have reported that knee instability can be considered as an important factor in motion predicting strategies for KOA patients during their walking [5]. Consequently, there is a possibility of having a kind of compensatory strategy in walking pattern for KOA patients with KI, which in turn will affect the disease progression [7]. In fact, knee instability may result in increasing joint’s movements in sagittal and frontal planes while walking and weight bearing, and altering the loading on the knee joint [1, 8]. This problem can affect the patients’ quality of life by reducing trust in the joint, and abstaining from daily activities [9, 10]. As a consequence, adding the joint instability to the knee osteoarthritis outcomes can put patients at fall risk, result in secondary problems, and also may change their walking pattern [11]. Evidence indicates increase in pain and even joint deformation in patients with early osteoarthritis subsequent to the improvement of instability in their knee [12]. Therefore, the emergence of KI as an accelerator in the arthritis symptoms recovery has recently caught the attention of specialists and researchers.

Total Resistance eXercises (TRX) is the new sling training for an intense full-body workout by which body coordination and stability can be improved effectively. The results of earlier studies have maintained that TRX exercises can activate the stabilizing muscles of various joints of the body, especially the core muscles that have the function of improving the lower extremity function [13]. Besides, the following advantages of this exercise are worth mentioning: its practicality for conducting variety of exercises, its attractiveness, simplicity, ease of use, and little space occupation [14]. Carrying out a study on TRX exercises, Bryan et al. (2014) reported that performing such exercises could increase the activation level of abdominal muscles [15]. Similarly, therapeutic exercises in water environment have been reported to be effective in improving proprioception and neuromuscular control. According to the previous literature on the issue, one of the best treatment protocols for people with knee osteoarthritis is water-based therapeutic exercises. Water properties such as hydrostatic pressure and water temperature can facilitate blood circulation. Also, water resistance that acts in the opposite direction to body motion may enhance muscular strengthening. Besides easing blood flow and promoting strength of muscles, specialists in the field have made reference to weight loss as a consequence of buoyancy force and pain receptors inhibition [16]. To add more, some studies have been conducted on the impact of aquatic exercises on pain and function in patients with knee osteoarthritis. To name a case, Alcalde et al. (2017) reported such advantages as reduced pain intensity, increased flexibility, improved functional capacity and quality of life following a 12-week aquatic physical therapy. Lu et al. (2015) also reviewed the effect of aquatic exercise on KOA patients. They concluded that aquatic exercise was effective and safe enough to be considered as an adjuvant treatment for patients with knee OA [17, 18]. Knee proprioception improvement has also been reported in patients with knee osteoarthritis following aquatic exercises. So, it is possible for the proprioception improvement, which is important in neuromuscular control, to affect joint dynamic stabilization and joint stability.

However, despite plethora of studies conducted on the risk factors of the knee joint instability in patients with osteoarthritis, to the best of researchers’ knowledge, no investigation has been conducted on its control strategies. Most of the therapists have just concentrated on the patients’ pain and function improvement, while neglecting knee joint instability. Prior to the present study, one study was conducted comparing some variables such as (Western Ontario and McMaster Universities Osteoarthritis Index (WOMAC), pain, gait pattern, Berg balance scale (BBS), function, and quality of life (SF_36_) between subjects with (*n* = 31) and without (*n* = 37) self-reported knee instability. The results manifested that KI could affect BBS (*p* = 0.016), function (p = 0.016), WOMAC stiffness subscale (*p* = 0.004) significantly [19].

The present study, subsequent to the results of the authors’ previous study was an attempt to look for appropriate interventions to reduce knee instability and co-existent factors in patients. The main purpose of the present study was to compare the aquatic and TRX exercises effects on the self-reported KI and its affected factors like balance, pain, WOMAC stiffness subscale, and also knee flexion ROM, quadriceps strength. Therefore, it was hypothesized that 1). TRX and aquatic exercises will improve pain, balance, stiffness, knee flexion ROM, quadriceps strength, and self-reported KI statistically, 2). the TRX would have more effectively reduced the self-reported KI than aquatic exercises intervention.

## Methods

### Trial design

**Fig. 1:**
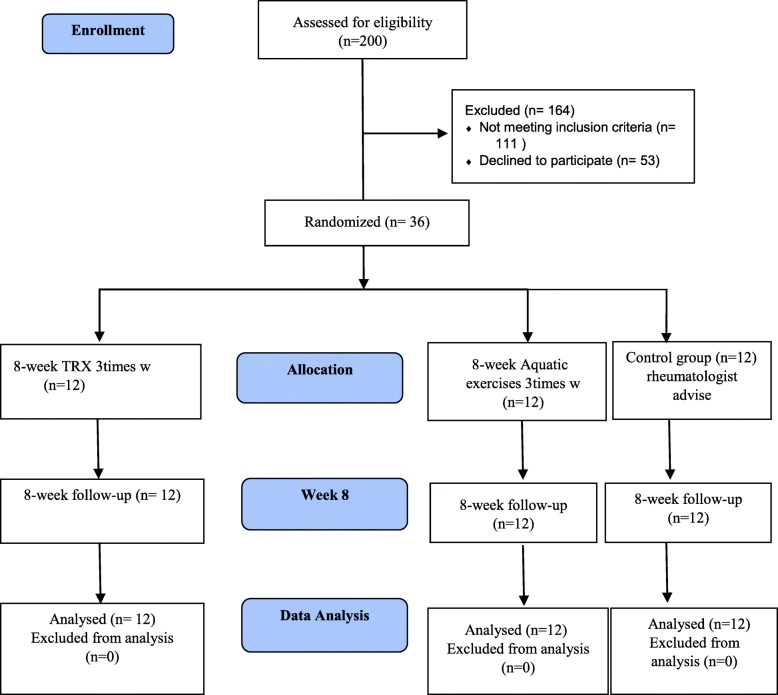
Participants Flow Diagram

This single blind, randomized, and controlled trial, which was conducted at Razi University rehabilitation center in Kermanshah, Iran, lasted for eight weeks starting in February and ending in May, 2019. The assessors who measured variables for patients were blinded about the group allocation. This study included three steps: 1. Pre-intervention measurements 2. 8-week aquatic and TRX exercises for case groups, and the control group which just received drug regimens by a rheumatologist, and 3. Post-intervention measurements (Fig. 1).

### Participants

A total of 200 knee osteoarthritis’ female patients were studied for eligibility among which 36 individuals were included in the present study. Of the 36 participants, 12 patients were allocated to the aquatic exercise, twelve to TRX exercises, and the rest to the control group. However, 111 patients didn’t meet the eligibility criteria and 53 declined to participate. The participants of the present study consisted of individuals from two groups: individuals who had regular referral from rheumatologist, and patients who waited for common physiotherapy in rehabilitation centers.

The criteria for including participants in the study were as follows: (1) having the age of more than 40 years, (2) acquiring the American College of Rheumatology clinical criteria for knee OA (Altman et al., 1986), (3) meeting the Kellgren and Lawrence radiographic disease severity scale ≥ II, and (4) self-reporting knee instability. Despite the abovementioned conditions, the participants were excluded in case they (1) had strokes, (2) faced uncontrolled hypertension, (3) were unable to walk without assistant instruments, (4) had received other treatment interventions in the past three months, (5) were suffered obesity (BMI > 40 kg/m^2^, 6) suffered from neuromuscular diseases like multiple sclerosis or Parkinson, (7) had lower extremity fracture, (8) afflicted with concurrent hip osteoarthritis, (9) waited for arthroplasty, (10) or cardiovascular diseases [7, 20]. Further, the patients who had received injection in the 6-month-period adjacent to the study, or had had surgical procedures were excluded from the study. Although the patients included in this study had bilateral arthritis, only the knee in which they reported symptom of instability was assessed. It is worthy to add that all the participating patients received the same drug regimens including Meloxicam 7.5 mg, Glucosamine Sulfate 750 mg, and Calcium-D in daily manner.

The G. Power (Ver. 3.1, Heinrich Heine University, Düsseldorf, Germany) software was used to estimate the minimum sample size. According to the reported results of previous researches and based on the test power of 0.90, the effect size of 0.63 and the significance level of 0.05, the minimum sample size was determined to be 36 [21]. Finally, the participants were randomly distributed into three groups, including TRX (*n* = 12), aquatic exercises (n = 12), and control (n = 12) (Fig. 1).

### Study interventions

#### TRX exercises

The TRX® Rip Trainer™ (China) was used for performing the exercises. TRX training was conducted by a TRX specialist, while an assistant coach also collaborated to prevent the patients from doing wrong exercises. The safety points were checked by a trainer in every session to avoid injuries before the exercises started. The TRX straps hanging down from anchor point, and Suspension Anchor™ were adjustable to execute various exercises. Exercises were designed based on the patients’ motion limitations like knee flexion and extension. Furthermore, TRX exercises were started at their most convenient forms while they gradually, but progressively, turned to be more and more difficult. The difficulty level of exercises increased through a step by step process by 1) Narrowing the base of support which increased the difficulty by reducing the stability 2) Changing the angle of pull, in other words, the farther the person was from the vertical, the greater the resistance 3) employing the pendulum in ground exercises in which the feet were placed in the Suspension Trainer and the hands were off the ground (head or back was on the ground). The gravity center in relationship to the perpendicular gravitational pull determined the difficulty. 4) and adding a handle which can increase the difficulty level of exercises. Each session time length was divided as: 5–10 min of each was allocated to introducing the sessions’ regarded exercises and their related correct techniques, 5–10 min were devoted to warm-up, which mainly included stretching exercises, and the remaining 40–50 min were regarded for performing the TRX exercises. Participants who were recognized with wrist pain through conducting the planks’ tests were allowed to put their forearm on the ground in order to prevent the wrist pain increasing. The TRX exercises protocol lasted for 60 min each session and was performed three times a week for eight weeks, 24 sessions altogether. The majority of the exercises were focused on the core muscles, hip abductors, and leg muscles strengthening (Table 1) [22].

**Table 1:** TRX-based exercises program

First Month	Exercises
**Sunday’s exercises**	1) TRX row, 2) TRX biceps curl, 3) TRX scapular retraction, 4) TRX standing roll out, 5) toe touches, 6) TRX hip press, 7) TRX hamstring curl, 8) walking high kick, 9) TRX Sit Up Plank exercises 3 set 10 s
**Monday’s exercises**	1) TRX mid row, 2) TRX calf raise, 3) TRX kick back, 4) TRX standing push up plus, 5) clamshell, 6) lying side leg lift/ lateral raise, 7) Hamstring runner TRX, 8) TRX bent raise (single leg), 9) TRX side plank. Plank exercises 3 set 15 s
**Wednesday’s exercises**	1) TRX high row, 2) TRX single leg reaching Roman deadlift, 3) TRX split fly, 4) TRX chest press, 5) lying leg raise, 6) TRX Routain, 7) supine plank TRX, 8) TRX bent leg raise, 9) TRX hip abduction. Plank exercises 3 set 20 s
At the week1 and 2, All exercises 3 set 10 repetition.	
At the week 3 and 4, All exercises 3 set 15 repetition.	
**Second Month**	**Exercises**
**Sunday’s exercises**	1) TRX T deltoid fly, 2) TRX standing hip drop, 3) TRX triceps press, 4) TRX standing calf raises 5) Flutter kicks 6) Side crunch leg raises 7) TRX supine plan/with pull through 8) TRX hip abduction, 9) TRX assisted sit up Plank exercises 3 set 20 s
**Monday’s exercises**	1) TRX Y deltoid fly TRX hip press, 2) TRX torso rotation, 3) TRX overhead back extension, 4) TRX prone iron cross, 5) Side oblique crunch, 6) Swimmers, 7) supine TRX on elbow, 8) TRX saw 9) TRX oblique leg raises Plank exercises 3 set 25 s
**Wednesday’s exercises**	1) TRX L deltoid fly, 2) TRX power pull 3) TRX bicep revers curl 4) TRX chest fly 5) Russian twist with medicine ball 6) Alternate heel touchers 7) TRX side plank/ top arm assisted pike 8) TRX pendulum, 9) TRX Pike Plank exercises 3 set 30 s
At the week1 and 2, All exercises 3 set 10 repetition.	
At the week 2 and 4, All exercises 3 set 15 repetition.	

#### Aquatic exercises

Aquatic exercise intervention was performed for eight weeks, three times a week, 24 sessions in total, with each session lasting for exactly 90 min. In other words, each participant was required to attend 24 sessions of rehabilitation with 90 min of duration for each session during the conducting phase of the study. The water temperature was approximately 32 °C (89 ° F), and the minimum water depth was considered 1.3 m. The water based exercises protocol included: 10 min of warm-up along with walking (forward, backward, and sidewalk), and also stretching exercises for lower extremity muscles (quadriceps, hamstrings, triceps surae, abductors and adductors of hip, and gluteal muscles), 20-min strength exercises with elastic band and sandbag (gluteus, adductors and abductors of hip, quadriceps, hamstrings, and triceps surae muscles); 20 min of aerobic exercises (stationary running or deep water-running); 20 min of step training and proprioceptive exercises; 10 min of core exercises, and finally 10 min of cool down. Based on the previous study findings, we selected exercises of the current study with the purpose of function, pain, and balance improvements [23, 24]. The aquatic exercises protocol was supervised by a certified physiotherapist in the pool (Table 2).

**Table 2:** Aquatic exercise program ^a^

Type of exercise	Exercises	Set
Warm up	walking (forward, backward, sidewalk, with kickboard) stretching exercise for lower extremity muscles: quadriceps, hamstrings, triceps surae, abductors and adductors of hip and gluteal muscles	3.10 (first 4 weeks) and 10 s rest 3.12 (second 4 weeks) and 10 s rest
Strength	Hip flexion, extension, and hyperextension, Hip abduction and adduction Knee flexion and extension, Double-Leg Calf Raise, Single-Leg Calf Raise, resisted hip extension, resisted hip abduction (resistance was considered water, noodle, and sand bag	3.10 (first 4 weeks) and 10 s rest 3.12 (second 4 weeks) and 10 s rest
Aerobic	Bounce: Knee lift/knee-high jog, Inner thigh lift/ankle reach Front, Leg curl/hamstring curl/heel-high jog, Kick front /straight leg Kick front/karate, Kick corner, Kick across, Kick side, Kick back, Cross-country ski, Bike on the noodle Jumping jack, Cross-country ski, Leap, Jazz kick/front, Jazz kick/corner, Pendulum	3.12 (first 4 weeks) and 10 s rest 3.15 (second 4 weeks) and 10 s rest
Step and proprioceptive	Gait training in anteroposterior, lateral-lateral, and diagonal. Then they will go up and down step alternating legs. Hand on hip as leg perform a rocking horse. Knee chest (supine, prone, and standing) Cross-country ski. Also, step up and step down: forward and side ward.	3.10 (first 4 weeks) and 10 s rest 3.12 (second 4 weeks) and 10 s rest
Core training	Stand and abduct and adduct the shoulder, Spinal rotation, standing with diagonal movement of hands with sand ball, Spinal rotation with sand ball, Bike on the noodle	3.10 (first 4 weeks) and 10 s rest 3.12 (second 4 weeks) and 10 s rest
Cool down	Deep breathing-forward and back ward tandem walking-static stretching interspersed with water walking- - Figure 8 arm sweep with spinal rotation and shoulder abduction and adduction.	10 s for each stretch

^a^ Random selection of exercises from the following list was performed during each session

### Randomization and blinding

Participants were randomized by the use of Random Number Generator Software (Research Randomizer, version 3.0), and also were allocated to three groups using Sequentially Numbered Opaque Sealed Envelopes (SNOSE) concealed allocation method. A physiotherapist who did not involve in the data collection and evaluation of the outcomes did the random allocation sequence, and enrolled as well as assigned participants to groups by random (allocation ratio 1:1:1).

The assessors of this research were blinded about the exercises and interventions assigned to the groups, but there was no possible way for blinding the subjects to training as well as the statistician towards the groups and their assigned exercises.

### Study outcomes

The current study was planned based on authors’ previous study and literature results [2, 6, 7]. In this line, the effect of 8-week aquatic and TRX exercises on factors that could be affected by KI including WOMAC stiffness subscale, balance, pain, KI, quadriceps strength and knee flexion ROM were evaluated.

#### Knee pain

Knee pain intensity was measured by employing a 10-cm visual analog scale (VAS) with the scoring range of 0 to10cm, in which “0” represented absence of any pain, “1” minimal pain, and “10” extreme or intolerable pain. To assess the intensity of knee pain of the participants, they were asked the following question: *“how much pain do you have during your daily activities?”* The VAS was used to measure the intensity of participants’ subjective pain prior to and after the interventions. A good reliability and validity has been reported for the VAS (ICC = 0.92) [25].

#### WOMAC stiffness

WOMAC is a reliable and valid instrument in the literature (ICC:0.80). The stiffness subscale consisted of two items based on the five-point Likert scale of 0 indicating no symptoms to 4 indicating extreme symptoms. The maximum score based on the scale was 8, (two four- point items) and the total range was from 0 to 8, with the zero indicating no symptom and 8 indicating the worst symptoms [26].

#### Berg balance scale (BBS)

Berg Balance Scale, which consisted of 14 different tasks, was used to assess balance in sitting and standing position and in transfer. Each motor task was rated by the use of a 5-point scale ranging from 0 to 4. The total score ranges from 0 to 56, where 56 represents normal balance. The test-retest reliability for the BBS was reported to be excellent (ICC = .71 to .99) [27].

#### Knee instability

Self-reported knee instability was evaluated according to giving way, and also shifting evidence, during the last month by Felson’s questionnaire [28]. Knee instability severity was graded based on the numerical scale (0 to 5) in response to the following question. The question was “What degree of giving way, buckling, or shifting of the knee would affect your daily routine activity?” The ratings were as follows: 5 = “I have no symptom”, 4 = “I have symptom, but it does not affect my ADL”, 3 = “Symptoms affect my ADL slightly”, 2 = “symptoms affect my ADL moderately”, 1 = “symptoms affect my ADL strongly”, 0 = “symptoms prevent me to perform all my everyday activities” [24]. The test-retest reliability of this self-report rating of KI was estimated by the use of an intra-class correlation coefficient (ICC =0.72) [29].

#### Knee flexion ROM

The Bubble inclinometer device (Fabrication Enterprises, Inc., White Plains, NY, USA) was used to measure Knee flexion ROM. The subjects were placed in prone position. Then the inclinometer was placed at the back of the tibia. The test was conducted on the limb which was more affected. Knee flexion was stopped in end-range of passive motion and further movement was restricted by pain. Three trials were recorded and the average of the three values was used for analysis [30].

#### Knee extensors strength

Maximal isometric strength of the knee extensors (quadriceps muscle) was measured using the Baseline Pull-Push Dynamometer (Model 12–0343, Fabrication Enterprises Inc., NY, USA). This digital dynamometer measures the force up to a maximum of 199.9 kg. Measurements were performed at 80°- 90° of knee flexion. The instrument was calibrated according to the instructions, before any measurement. The patients were seated in a comfortable position with the backrest angled at 100°. The shin pad was placed 2 cm above the medial and lateral malleoli. The instrument shaft remained horizontal to the anterior aspect of the mid shaft of tibia and horizontal to the posterior aspect over the musculotendinous junction of calf muscles. Subjects were then asked to remain at that position while pushing against the dynamometer. Also, the subjects were required to push against the gauge pad as hard as possible when given the appropriate command. All measurements were performed with the limb segment in a position that was with gravity eliminated. Resting times between trials were approximately 60 s. Each contraction was held for six seconds. The peak force was recorded and average of records was considered as the quadriceps strength [31].

### Ethical considerations

This study’s protocols were reviewed and approved by the research ethics committee of the Medical Sciences University of Kermanshah in Iran (Registration no.: IR.UMMS.REC.1397.718). The study’s protocol was also registered in the Iranian Registry of Clinical Trials (Registration no.: IRCT20181222042070N1). All the tests and measurements were carried out at the Sport Rehabilitation Laboratory of Razi University, Iran. As well, all the participants were provided with related informed consent forms which were both completed and signed by participants in person.

### Statistical analysis

We analyzed balance, Pain, WOMAC (stiffness), knee flexion ROM, quadriceps strength, and self-reported knee instability variables before and after the 8-week aquatic and TRX exercises.

First, we used Shapiro-wilks and Leven’s test for assessing the normal distribution of data, and also the variances homogeneity. When variances normality and homogeneity tests were confirmed, the data were considered to be parametric. Consequently, demographic and baseline parameters were analyzed by the one-way analysis of variance (ANOVA). Additionally, Tukey’s post hoc test was used for the pairwise comparisons. In order to compare the changes of each dependent variable over the time (t0 = pretest, and t1 = posttest) and between groups, the variables were analyzed by employing mixed-model repeated measures ANOVA using time and group as factors time × group (2 × 3). In the presence of significance, Tukey’s post hoc test pairwise comparisons were used. Also, we used Paired samples t-test for pretest to posttest assessing in each group. Statistical analysis was performed using statistical software, SPSS version 20.0 (IBM SPSS, Armonk, NY, USA). Statistical significance was determined at *p*-values less than 0.05. All results were reported as the mean ± standard deviation.

## Results

The results of Shapiro Wilks and Levine’s test indicated that both assumptions for data distribution normality and homogeneity of the variances were accepted (*P* > 0.05). The obtained comparative results about demographic characteristics and dependent variables in the baseline for interventions and control groups are displayed in Table 3. The results of one-way ANOVA and Tukey’s test indicated that there were no significant differences between the treatment and control groups in the baseline characteristics (all P > 0.05).

**Table 3:** Demographic, anthropometric and pre-intervention knee instability-related characteristics of study participants

Variables	TRX (*n* = 12) M (SD)	Aquatic exercises (*n* = 12) M (SD)	Control (*n* = 12) M (SD)	P-value^a^
Age _(year)_	55.9 (8.6)	57**.**5 (6.9)	63.8 (7.5)	0.08
Weight _(kg)_	80.9 (3.4)	78.2 (10.9)	73.6 (8.9)	0.3
Height _(cm)_	161.9 (5.7)	165.6 (6.8)	162.5 (4.7)	0.058
BMI _(kg/m2)_	29.8 (7.2)	28.5 (3.7)	23.1 (11.6)	0.07
VAS_(cm)_	6.8 (2.4)	7.2 (2.2)	8.3 (2.2)	0.3
Kellgren & Lawrence _(grade 1–4)_	2.7 (0.8)	3.0 (0.6)	2.7 (0.6)	0.6
Knee Instability Score _(0–5)_	2.1 (1.6)	1.6 (1.4)	2.9 (1.4)	0.1
Stiffness Score _(0–8)_	4.4 (2.3)	4.9 (2.1)	4.7 (1.2)	0.8
VAS_(cm)_	7.6 (2.4)	7.7 (2.1)	7.5 (2.0)	0.8
BBS Scores _(0–56)_	37.6 (8.9)	41.3 (8.3)	37.9 (8.3)	0.4
Quadriceps strength _(Kg)_	13.4 (1.6)	14.9 (1.6)	12.7 (1.7)	0.6
Knee flexion _(°)_	104.7 (6.5)	110.4 (6.4)	115.7 (6.9)	0.5

^a^ No significant differences among groups for pretests. *BMI* Body Mass Index, *SD* Standard Deviation, *BBS* Berg Balance Scale, *VAS* Visual Analog Scale, *WOMAC* Western Ontario and McMaster Universities Osteoarthritis

### Self-reported KI

**Fig. 2:**
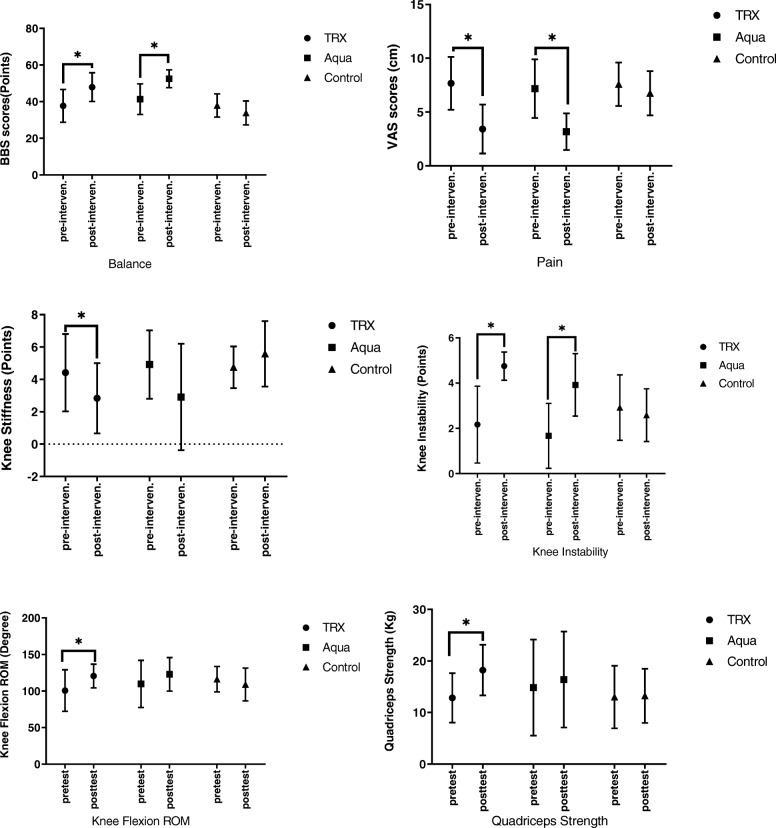
Pretest to posttest mean comparison for TRX training, aquatic exercises and control groups about studied variables. (*) statistical significant differences at the 0.05 level

Regarding the interaction effect of time × group (*P* = 0.0001), Instability scores were significantly reduced over time in both aquatic and TRX exercises (*P* < 0.0001). In both intervention groups, a significant improvement in instability scores was detected from 8 weeks compared to the baseline (t0 vs. t1, P = 0.0001 in TRX; t0 vs. t1, P = 0.0001 in aquatic exercises), but this was not significant in control (t0 vs. t1, *P* = 0.45). Additionally, the differences amongst groups regardless the time were significant (*P* = 0.02). Moreover, Tukey’s post hoc test indicated that there was no significant difference between aquatic exercises and TRX (*P* = 0.84), but there was a significant difference between the aquatic exercises and the control (*P* = 0.03) as well, there was significant difference between TRX and control groups (*P* = 0.04) (Fig. 2, Table 4).

**Table 4:** Changes in clinical outcomes after 8-week TRX and aquatic interventions

Outcome measure	groups	t0 M (SD)	t1 M (SD)	Change by the time	Between group difference	AQ vs. TRX	AQ vs. CON.	TRX vs. CON.
Knee Instability	TRX	2.1 (1.6)	4.7 (0.6)	0.0001^a^	0.02^c^	0.84	0.03^b^	0.04^b^
	Aqua therapy	1.6 (1.4)	3.9 (1.2)	0.0001^a^				
	Control	2.9 (1.4)	2.5 (1.1)	0.45				
WOMAC Stiffness	TRX	4.4 (2.3)	2.8 (2.1)	0.04^a^	0.12	0.92	0.25	0.05^b^
	Aqua therapy	4.9 (2.1)	2.9 (0.02)	0.058				
	Control	4.7 (1.2)	5.5 (2.0)	0.14				
VAS	TRX	7.6 (2.4)	3.4 (2.2)	0.0001^a^	0.03^c^	0.63	0.03^b^	0.04^b^
	Aqua therapy	7.1 (2.7)	3.1 (1.6)	0.0001^a^				
	Control	7.5 (2.0)	6.7 (2.5)	0.13				
BBS	TRX	37.6 (8.9)	47.91 (7.88)	0.0001^a^	0.0001^c^	0.92	0.001^b^	0.10
	Aqua therapy	41.3 (8.3)	52.5 (4.88)	0.0001^a^				
	Control	37.9 (2.2)	33.83 (1.88)	0.45				
Quadriceps strength	TRX	13.4 (1.6)	16.9 (2.6)	0.001^a^	0.42	0.80	0.21	0.31
	Aqua therapy	14.9 (3.5)	16.4 (4.3)	0.21				
	Control	12.7 (4.7)	12.2 (5.7)	0.88				
Knee Flexion	TRX	109.7 (5.5)	129.4 (4.9)	0.03^a^	0.05	0.56	0.61	0.90
	Aqua therapy	110.4 (6.7)	121.9 (8.5)	0. 10				
	Control	115.7 (8.9)	110.2 (5.2)	0. 12				

*SD* Standard Deviation, *VAS* Visual Analogue Scale, *WOMAC* Western Ontario and McMaster Universities Osteoarthritis Index, *BBS* Berg Balance Scale; t0: baseline measures; t1: 8-week measures

(^a^) p < 0.05 for posttest compare to the baseline. (^b^) means p < 0.05 for post hoc pairwise comparison between groups. (^c^) p < 0.05 between groups differences

### Pain

Regarding the interaction effect of time × group (*P* = 0.001), VAS scores for pain significantly decreased over time in both TRX and aquatic groups (*P* < 0.0001). In both intervention groups, a significant improvement in VAS scores were detected from 8 weeks compared to the baseline (t0 vs. t1, *P* = 0.0001 in TRX; t0 vs. t1, P = 0.0001 in aquatic exercises), but this was not significant in control group (t0 vs. t1, *P* = 0.13). Additionally, the differences amongst groups regardless the time factor were significant (*P* = 0.03). Tukey’s post hoc test indicated that there was no significant difference between the aquatic exercises and TRX (*P* = 0.63), but on the contrary, there was a significant difference between the aquatic exercises and control groups (P = 0.03). Also, there was significant difference between the TRX and control (*P* = 0.04) (Fig. 2, Table 4).

### WOMAC (stiffness)

Regarding the effect of time × group (*P* = 0.023), stiffness subscale scores of WOMAC significantly improved over time (P = 0.04). A significant improvement in stiffness scores was detected from 8 weeks compared to the baseline (t0 vs. t1, P = 0.04 in TRX), but this was not significant in aquatic and control groups (t0 vs. t1, *P* = 0.058, t0 vs. t1, *P* = 0.14). Additionally, the differences amongst groups regardless the time factor weren’t significant (*P* = 0.12 in control). Tukey’s post hoc test, also, showed that there wasn’t any significant difference between aquatic exercises and control groups (*P* = 0.25). However, there was a significant difference between TRX and the control (P = 0.05), and there was still no significant difference between the aquatic exercises and TRX (*P* = 0.92) (Fig. 2, Table 4).

### Berg balance scores

Regarding the interaction effect of time × group (*P* = 0.0001), balance scores were significantly increased over time (*P* < 0.0001). A significant improvement in balance scores were detected from 8 weeks compared to the baseline (t0 vs. t1, P = 0.0001 in TRX; t0 vs. t1, P = 0.0001 in aquatic group), but this was not significant in control group (t0 vs. t1, *P* = 0.45). Likewise, the differences between groups regardless the time factor were significant (P = 0.0001). As Tukey’s post hoc test indicated, there was a significant difference between the aquatic exercises and control groups (*P* = 0.001). There was a non-significant difference between the TRX and control (*P* = 0.10), but there was no significant difference between the aquatic and TRX groups (*P* = 0.92) (Fig. 2, Table 4).

#### Knee flexion ROM

Regarding the interaction effect of time × group (*P* = 0.031), a significant improvement in knee flexion scores were detected from 8 weeks compared to the baseline (t0 vs. t1, P = 0.03 in TRX), but this was not significant in aquatic and control groups (t0 vs. t1, P = 0.1 in aquatic, and *P* = 0.12 in control). Further, the difference between groups regardless the time factor was significant (*P* = 0.05) As Tukey’s post hoc test indicated, there wasn’t significant difference between the aquatic exercises and control groups (*P* = 0.61). There wasn’t a significant difference between the TRX and control (*P* = 0.90). also, there was no significant difference between the aquatic and TRX groups (*P* = 0.56) (Fig. 2, Table 4).

### Quadriceps strength

Regarding the interaction effect of time × group (*P* = 0.028), quadriceps strength scores also significantly increased over time (*P* = 0.01). A significant improvement for quadriceps strength scores were detected from 8 weeks compared to the baseline in TRX (t0 vs. t1, *P* = 0.001), but there was no significant improvement for aquatic and control groups (t0 vs. t1, *P* = 0.21; t0 vs. t1, *P* = 0.88). Additionally, the differences amongst groups regardless the time factor weren’t significant (*P* = 0.42) (Fig. 2, Table 4).

## Discussion

The results of the present study are in agreement with the first hypothesis of the study which states that there is no significant statistical difference in all the research outcomes between intervention groups except for the WOMAC (stiffness), which indicates a significant difference between TRX and control groups but no significant difference between aquatic and control groups. In fact, the improvement of the measures in the dependent variables subsequent to the aquatic intervention might be due to the following: 1. the water’s viscosity or resistance which can be very effective for muscle retraining as well as for increasing the rehabilitation progressions, 2. hydrostatic pressure, which supports and stabilizes the patients, allowing people with balance deficits to perform exercises without the fear of falling, 3. water warmth, which can lead to reduction in pain and muscle spasm, 4. buoyancy, which decreases loading of joints, and finally the unique characteristics of water-based exercising which may allow people to perform exercises which otherwise they would be unable to perform on land. The findings of some of the studies in the field including Alcalde et al. (2017), Taglietti et al. (2018), and Lu et al. (2015) are in line with our findings [17, 18, 24]. Consistent with the results of the present study, they reported that aquatic exercises could improve pain, function, and balance in patients with KOA. However, WOMAC (stiffness) outcome wasn’t significant between aquatic and control groups. It may be the result of non-significant improvement (pretest to posttest) in quadriceps strength for aquatic group. This is in corroboration with the findings of studies reported recently, indicating that the presence of self-reported KI may be a sign of inadequate dynamic knee joint stability and diminished knee joint control in the patients’ population [1, 7]. Quadriceps muscle is one of the important dynamic knee joint stabilizers, so the inadequate quadriceps strength can cause the higher rate of knee joint instability in knee osteoarthritis patients with KI [7]. Moraiti et al. (2009) have also reported that deficits in muscular strength and proprioceptive sensory input, which are thought to alter the neural control of the muscles around the knee joint, are associated with greater knee flexion/extension motion variability after ACL reconstruction [32, 33].

Moreover, the findings of the present study indicated the effectiveness of the TXR intervention as a significant improvement of the dependent variables. Body weight and TRX sling provide an appropriate resistance to strengthen the core and extremities muscles in KOA patients. Both intervention protocols had strengthening exercises in order to strengthen the core muscles, as well as the thigh and the leg muscles. The outcomes, however, indicated that quadriceps muscle strength increased significantly from pretest to posttest just for TRX group. This different strength progression between groups maybe due to muscle relaxation caused by the increased water temperature. Significant improvements in pain, balance, WOMAC (stiffness), quadriceps muscle strength, knee flexion ROM, and self-reported KI subsequent to TRX intervention could have been due to the strengthening of the core and leg muscles. This is in accordance with Foroughi et al. (2019) who reported that adding isolated core postural control training to physiotherapy exercises was noticeably associated with greater improvements in pain, function, and center of pressure trajectories than physiotherapy exercises alone [34]. Likewise, Arazi et al. (2018), and León et al. (2019) study corroborated the same findings. They suggested that extremities function was influenced by lumbo-pelvic-hip muscular strengthening in TRX exercises [35, 36]. In line with the findings in the literature and consistent with our findings, it seems that TRX exercises could strengthen the hip and core muscles to the extent that the patients can put the foot within the base of support area, resulting in confidence improvement in patients. This is consistent with Shakoor et al. (2017), which found that quadriceps muscle strength can be an important predictor for worsening the knee instability [37]. Reduction in the dynamic KI could decrease pain and knee stiffness. Subsequently, reduced knee stiffness could improve knee ROM and balance in KOA patients.

With regard to the second hypothesis of the study, significant statistical differences in all study outcomes were found from pretest to posttest in intervention groups except for WOMAC (stiffness subscale), quadriceps muscle strength, and knee flexion ROM. Additionally, the differences were significant between TRX and control groups. However, no significant difference in aquatic and control groups for WOMAC stiffness was reported. It seems that TRX exercises could reduce compensatory knee joint-stiffening strategy by significantly strengthening the dynamic knee stabilizer muscles including quadriceps. Besides, it can improve dynamic stability and neuromuscular control, ultimately improving the painless knee flexion ROM. The above findings are consistent with Dixon et al. (2010) who reported that greater self-reported stiffness was associated with lower peak knee adduction moment for the OA patients [38]. Esch et al. (2006) also reported that patients with OA, high knee joint laxity, and low muscle strength are most at risk of being disabled [39]. Similarly, Zwart et al. (2015) reported the same results. They assessed the associations between knee muscle strength and falls, controlling for knee joint proprioception, varus-valgus knee joint laxity, and knee instability among patients with knee osteoarthritis, who reported knee instability. They draw the conclusion that high knee extension and flexion muscle strength decreased the risk of falls in patients with knee OA and self-reported knee instability [40].

Some researchers have conducted comparable studies on aquatic-based and land-based exercise effects on function, mobility and other health outcomes in people with knee and hip osteoarthritis. They reported the same results for aquatic exercise for adults with arthritis as those of land-based exercises [41, 42]. Likewise, Wyatt et al. (2001) conducted a study to detect differences between an aquatic exercise program and a land-based exercise program on KOA patients’ pain and function. They reported that both aquatic and land-based exercise programs are beneficial to patients with osteoarthritis [43], the findings which are in corroboration with the results of our study. On the other hand, Lund et al. (2008), compare the efficacy of aquatic and land-based exercise program in patients with knee osteoarthritis. They concluded that land-based exercise showed some improvement in pain and muscle strength compared to the control group, while no clinical benefits were detectable from aquatic exercise [44].

Due to the study setting and extraneous variables, the present study could not be free from its own limitations. First the analyses were based on the self-reports of instability symptoms instead of instability objective measurements. Second small sample size and inability to use more groups can be regarded as another limitation; however, the authors have justified the number of the patients required to participate in their study by calculating the statistical power. With regard to the third limitation of the study, one can highlight the sex of participants (as only women participated in the study). The fourth limiting factor can be the failure to control the daily diet of patients which can in itself affect the joint’s health as well as the subjects’ life style. Ultimately, the participants’ motivation regarding how they do exercises as a control variable might result in different results. In the future, it may be worthwhile to examine the mixed model of TRX-aquatic exercises, and compare them with the TRX and aquatic exercises. Additionally, it may be worthy to examine the Pilates and TRX intervention effect on other variables like quadriceps strength and muscle EMG.

## Conclusion

Based on the study findings, the conclusion was drawn that TRX and water-based interventions had a similar effect on the self-reported KI, pain, and balance variables. However, compared to water-based exercises, TRX exercises had more effect on knee stiffness, quadriceps strength, and knee flexion ROM. As a result, TRX intervention could be recommended to physical therapist as an appropriate protocol for the KOA patients rehabilitation.

## Data Availability

The datasets used and/or analyzed during the current study are available from the corresponding author on reasonable request.

## Abbreviations

KOA: Knee osteoarthritis
TRX: Total Resistance eXercises
KI: Knee Instability
SNOSE: Sequentially Numbered Opaque Sealed Envelopes
ADL: Activity daily living
ROM: Range of Motion
BBS: Berg Balance Scale
WOMAC: Western Ontario and McMaster Universities Osteoarthritis Index
VAS: Visual Analog Scale
ES: Effect Size
EMG: Electromyography
BMI: Body Mass Index
ANOVA: Analysis of variance
